# Does financial inclusion help alleviate household poverty and vulnerability in China?

**DOI:** 10.1371/journal.pone.0275577

**Published:** 2022-10-14

**Authors:** Youxue Jiang, Yangyi Liu

**Affiliations:** 1 School of Economics, Nanjing University, Nanjing, China; 2 School of Finance, Jiangsu Vocational Institute of Commerce, Nanjing, China; 3 School of Economics, Si Chuan University, Chengdu, China; Szechenyi Istvan University: Szechenyi Istvan Egyetem, HUNGARY

## Abstract

This paper investigates the impact of financial Inclusion on household poverty and vulnerability by constructing a household financial inclusion index using the China Household Finance Survey 2015. It is found that financial Inclusion significantly reduces the probability of poverty and vulnerability of households and has a more significant impact on vulnerable groups such as rural and urban low-income people. Further, financial Inclusion has a more significant effect on poor families that do not receive government support for poverty alleviation and can complement co-insurance mechanisms to help families better cope with vulnerabilities caused by synergistic community shocks. Finally, promoting entrepreneurship and improving risk management capabilities are the main channels of financial Inclusion.

## 1. Introduction

Compared with static poverty indicators in a general sense, household poverty vulnerability portrays the likelihood of household poverty in the future and is a forward-looking indicator reflecting the dynamic changes of household poverty, which helps to represent and reflect the issue of poverty eradication quality. Financial poverty alleviation is a necessary support and guarantee for poverty eradication in China. Financial development can alleviate poverty through the indirect mechanism of promoting economic growth and the direct means of providing financial services [1–3]. However, problems such as financial volatility and backwardness of rural finance in the traditional economic development process inhibit the poverty-reducing effects of financial development [4–6]. As China’s economic growth slows, the incentive to rely on rapid economic growth to reduce poverty decreases and the trickle-down effect of economic growth is difficult to reach all groups [7]. In this context, financial services have become an essential mechanism foreconomic poverty reduction. Some scholars have considered microfinance as an essential way of economic poverty reduction by providing financial services to poor households, low-income groups, or MSMEs [8, 9], but the problems of business sustainability, inability to reach the abysmal and excessive debt burden of clients, have seriously limited the effect of microfinance on poverty reduction. It is debatable whether it is genuinely beneficial to the poor [10, 11]. [12] points out that microfinance should not be considered a highly effective solution to poverty, but other practical strategies for financial Inclusion should be actively pursued.

Financial Inclusion is an important initiative of China’s current economic reform. Unlike traditional economic development that overemphasizes the depth of financial services, financial Inclusion focuses on the breadth and outreach of financial services, aiming to provide appropriate and effective financial services at affordable costs to all social classes and groups in need of financial assistance. The comprehensive strength of China’s financial sector has increased significantly in recent years. However, the distribution of economic resources has not been fundamentally improved, and some disadvantaged groups still have difficulties receiving adequate financial support. According to the World Bank’s 2014 Financial Inclusion Survey, only 9.5% of adults in China have access to bank loans, while 20–30% of adults in developed countries such as the United States, Germany, and the United Kingdom have access to bank loans, and 11.6% and 6.2% of people in the top 60% and bottom 40% of China’s income have access to bank loans, respectively. To this end, the Chinese government has proposed the development of inclusive finance, and the "Implementation Opinions on Financial Assistance to Fight Poverty" issued by the People’s Bank of China and others also proposed to take the development of inclusive finance as the foundation and make every effort to promote financial services in poor areas to villages and households to people.

Whether financial Inclusion effectively reduces poverty in China is the core of this paper. In recent years, although domestic and foreign scholars have begun to pay attention to the poverty-reducing role of financial Inclusion, the research is not yet sufficient. First, the literature has studied chiefly the impact of financial Inclusion on poverty at the macro level, with a relative lack of micro evidence and insufficient evidence on the mechanism of financial Inclusion’s action in reducing poverty. For example [13, 14], found that financial Inclusion can significantly reduce poverty in India and Asian countries using data from India and Asian countries, respectively. Some literatures argue that financial inclusion has a particularly pronounced effect on poverty reduction in developing countries [15]. In addition, a sample survey of Indian households and a survey of poor households in Ghana show that financial inclusion can significantly reduce household poverty [16, 17]. Secondly, the literature has examined chiefly the impact of financial Inclusion on poverty only, while less incorporating vulnerability considerations, which is not conducive to studying the effects of financial Inclusion on the quality of poverty eradication. For example [18], points out that although traditional poverty alleviation measures are necessary, financial Inclusion provides new answers to address poverty. [19] describes the role of financial Inclusion in reducing poverty in various financial services such as savings, credit, insurance, and payments. Again, although a few pieces of literature have preliminarily explored the impact of financial Inclusion on vulnerability, the analysis of the mechanism of action of exposure and the sources of risk shocks is yet to be deepened. [20] theorizes the impact of financial Inclusion on household vulnerability and that the absence of financial Inclusion will increase the effects of risk shocks on household vulnerability. [21] studied the impact of financial Inclusion on rural household poverty vulnerability by constructing a village financial inclusion index. Finally, the literature has mainly studied the effects of financial Inclusion on rural poverty in China, while less incorporating urban poverty, which both ignores the lack of financial services and urban poverty among low-income groups in urban China and makes it challenging to inform China’s integrated urban-rural poverty governance in post-2020 poverty alleviation.

Given this, based on the China Household Financial Survey (CHFS) 2015 data, this paper studies the impact of financial Inclusion on household poverty and vulnerability in China and its mechanism of action by constructing a household financial inclusion index, and further analyzes what role financial Inclusion plays in poverty reduction, whether it can improve the effectiveness of poverty alleviation, and whether it can serve as a supplement to the co-insurance mechanism to help households effectively cope with synergistic vulnerability due to shocks. The main contributions of this paper are (1) incorporating poverty and vulnerability into a unified framework, analyzing not only the impact of financial Inclusion on poverty, but also examining the impact of financial Inclusion on vulnerability, and examining the role of financial Inclusion on the vulnerability of different sources of risk shocks through vulnerability decomposition. (2) The household financial inclusion index is constructed using microdata, and the mechanism of financial Inclusion’s impact on poverty and vulnerability issues is critically analyzed. (3) Urban poverty is added to study the impact of financial Inclusion on different groups’ poverty status, such as urban and rural areas, which is an essential reference for formulating and implementing China’s future poverty alleviation strategies. The rest of the structure is arranged as follows: the second part is data and variables, the third part is empirical analysis, the fourth part is impacted mechanism analysis, the fifth part is further analysis, and finally, the conclusion and policy implications.

## 2. Methods and data

### 2.1. Data

This paper uses the China Household Finance Survey (CHFS) 2015. The survey adopts a three-stage stratified sampling method proportional to the population size, and the sample is representative and of high data quality [22]. 2015 survey covers 29 provinces/municipalities/autonomous regions, 363 counties/districts/county-level cities, and 1439 communities/villages, except for Hong Kong, Macao, Taiwan, Xinjiang, and Tibet. It obtains microfinance data on 37300. The micro-financial data of more than 37300 households were acquired, including information on assets and liabilities, income and expenditure, insurance, and protection. They consider that urban families in China also have inevitable poverty and vulnerability problems. This paper studies rural and urban areas together to formulate and implement China’s future poverty alleviation strategy.

We removed the extreme value of 0.5% above and below the assets and income data. In addition, because in China, if the age of the head of household is less than 16 years old or above 80 years old, it is considered to be incapable of working, so this paper deletes the age of the head of household less than 16 or more than 16 years old. 80-year-old sample. It should be noted that there are 177 samples in the deleted data, accounting for 0.47% of the total number of samples 37,300. Therefore, it can be considered that deleting extreme values has a limited impact on the total number of samples and little impact on the empirical results. There are 49 samples with missing data, accounting for 0.13% of the total number of samples of 37300. We use the following method to fill in linear interpolation.

Linear interpolation is a relatively simple method, and its interpolation function is a first-order polynomial. Let the value of the function *y* = *f*(*x*) at the two points *x*_0_, *x*_1_ be *y*_0_, *y*_1_ respectively, and find the polynomial φ_1_(*x*_0_) = *y*_0_, φ_1_(*x*_1_) = *y*_1_,so that it satisfies.

From analytic geometry: $y=\varphi_{1}(x)=y_{0}+\frac{y_{1}-y_{0}}{x_{1}-x_{0}}(x-x_{0})$, From this we get:

$$\varphi_{1}(x)=f(x_{0})+f(x_{0},x_{1})(x-x_{0})$$

The above interpolation polynomial is a first-order polynomial, and this kind of interpolation is called linear interpolation.

### 2.2. Poverty and vulnerability indicators

This paper mainly uses the World Bank’s consumption poverty line. On the one hand, China only has a rural poverty line and lacks an urban poverty line; on the other hand; the literature points out that there are significant measurement errors in income data from micro surveys and that consumption data can be a more accurate measure of household welfare [23]. The World Bank in 2011 defined the extreme poverty line as $1.9 based on purchasing power parity and the poverty line based on median per capita consumption in other developing countries as $3.1. In addition, this paper uses the vulnerability indicator (Vulnerability as Expected Poverty, VEP) proposed by [24] based on the definition of expected poverty, where a household is vulnerable if its probability of experiencing poverty in the future exceeds a set vulnerability threshold. The VEP approach reflects changes in poverty dynamics, has the advantage of being forward-looking, applies to cross-sectional data, and has been widely used by scholars [25–28].

Starting from the definition of VEP, vulnerability can be expressed by a general formula.


$$V_{ht}=E[p_{ah,t+1}\;\left(c_{h,t+1}\right)\;\left|F(c_{h,t+1}|I_{T}\right)]$$

Where *V*_*ht*_ indicates the vulnerability of the hth household in period t, *c*_*h*,*t*+1_ indicates the welfare level (income or consumption) of the family in period t + 1, and *F*(*c*_*h*,*t*+1_|*I*_*T*_) indicates the distribution function of the welfare level in period t + 1. *I*_*T*_ shows the information provided by the panel data in period t. The implication of the above formula is clear: the magnitude of household vulnerability depends on the characteristics of the distribution of future levels of household welfare: the form of the distribution and the parameters of the distribution, which need to be estimated based on the information available *I*_*T*_.

About the form of the income distribution, [29]after a detailed study of the state of the income distribution, the Pareto distribution is a good fit for the upper middle income, especially for the wealthiest 20% of the population. The tails of the high-income component of the lognormal distribution are less dense than those of the Pareto distribution. They are better suited to describe the income distribution of the lower-income groups. [30] also uses the assumption of a log-normal distribution in their vulnerability study. Therefore, we assume that future income follows a log-normal distribution. In turn, the formula for measuring poverty vulnerability can be specified as:

$$V_{h}=\int \frac{\ln z}{-\infty }P_{\alpha }(\ln ch_{h+1})d(\ln ch,t+1)$$

For the poverty indicator $p_{\alpha }(c,z)={\left(\frac{\max(\ln z-\ln c_{h,t}+1,0)}{\ln z}\right)}^{a}$, we only consider the case where α = 0. and $\ln c_{h,t+1}∼N(\mu_{\ln c_{h,t+1}},\;\sigma_{\ln c_{h,t+1}}^{2})$.

After determining the form of the distribution of income, it is then necessary to estimate the mean and variance of the distribution, which can be complex or straightforward: firstly, according to Friedman’s theory of persistent income, long-term consumption expenditure depends on a steady income, which is a stable flow of income expected over a long period (more than three years) and can be calculated from the weighted average of income observations over several years, i.e., we can This means that we can directly calculate the intertemporal mean and variance of household income as the mean and variance of the future income distribution. However, the author’s study found that the lack of data for some of the income determinants in the CHNS is significant, making the regression-based vulnerability measure less efficient. Therefore, in this paper, the first approach uses the directly calculated inter-period mean and variance of household income as the mean and variance of the future income distribution.


$$\mu_{\ln c_{h,t+1}}=\frac{1}{T}\sum_{t=1}^{T}\ln c_{t}$$

$$\sigma_{{\mathrm{lnr}}_{lk+1}}^{2}=\frac{1}{T-1}\sum_{t=1}^{T}{\left(\ln c_{ht}-\mu_{\ln c_{h,t+1}}\right)}^{2}$$

It follows that vulnerability is a probability that measures the likelihood of experiencing poverty in the future, and a household is vulnerable if it exceeds a set vulnerability line. In the context of China’s poverty eradication efforts, we set the vulnerability line mainly based on the incidence of poverty [31, 32]. For example, combined with Table 1, if the vulnerability line is 1.9 USD, roughly 24.49% of households nationwide are vulnerable. Table 1 reports the results of poverty and vulnerability descriptive statistics. It can be seen that although absolute poverty has been improved in China, the proportion of vulnerable households is still at a high level, and changes in poverty groups need to be dynamically tracked in poverty eradication and future poverty alleviation practices; in addition, although poverty and vulnerability are more severe in rural China, they also exist to some extent in urban areas.

**Table 1:** Descriptive statistics of household poverty and vulnerability.

Region	Poverty rate		Vulnerability rate	
	1.9USD	3.1USD	1.9USD	3.1USD
National	0.0615	0.1429	0.2449	0.4982
Rural	0.1341	0.2967	0.3674	0.4348
Urban	0.0374	0.0788	0.1482	0.2605

### 2.3. Family financial inclusion index

We use factor analysis to construct a household financial inclusion index in five dimensions: having a bank account, access to formal credit, commercial insurance coverage, using digital financial services, and holding a credit card. On the one hand, the existing literature mainly constructs national or regional macro-financial inclusion indices [33–37]. Macro indices are difficult to directly reflect the distribution of financial resources among micro-individuals. They cannot accurately assess the micro-welfare effects of financial Inclusion because of the long geographical and economic distance from micro-households. [38] investigated the impact of financial Inclusion on household income by constructing a financial inclusion index.

On the other hand, bank accounts are the groundwork for financial Inclusion [39]. Moreover, insurance can provide risk protection and enhance the economic effects of loan services [40]. Considering the importance of digital financial development for financial Inclusion, we included digital financial services indicators [33]. Table 2 reports the results of the factor analysis. The KMO (Kaiser-Meyer-Olkin) test statistic is an indicator used to compare simple and partial correlation coefficients between variables. The KMO statistic takes on a value between 0 and 1. When the sum of the squares of the simple correlation coefficients between all variables is much greater than the sum of the courts of the partial correlation coefficients, the closer the KMO value is to 1, the stronger the correlation between the variables and the more suitable the original variables are for factor analysis; when the sum of the squares of the simple correlation coefficients between all variables is close to 0, the closer the KMO value is to 0, the weaker the correlation between the variables and the less suitable the original variables are for factor analysis. The first public factor was retained according to the standard criteria of eigenvalues more significant than one and a cumulative explanatory ratio greater than 75%, which can reflect the financial inclusion status of households. In this paper, the Bartlett factor score method is used to construct the financial inclusion index, and finally, the financial inclusion index is standardized to take values between [0, 100].

**Table 2:** Results of factor analysis.

	Eigenvalue	Cumulative explanation		KMO	Factor load
Factor1	2.1715	0.8420	Have a bank account	0.8050	0.6252
Factor2	0.2552	0.9450	Obtain a formal loan	0.7850	0.4462
Factor3	0.1450	0.9950	Have commercial insurance	0.8202	0.5326
Factor4	0.0152	1.0000	Use digital financial services	0.7520	0.7526
Factor5	-0.002	1.0000	Have a credit card	0.7263	0.8503

Note: The overall value of the KMO test is 0.7662, and digital financial services include Internet payment, finance, and financing.

### 2.4. Variable descriptive statistics

Table 3 reports the results of the descriptive statistics of the variables. In addition to the financial inclusion index, we control for other household characteristic variables in the regression model (net worth, income, share of financial assets, financial literacy, relationship network, private lending, social pension insurance, and balance, social health insurance, and balance, risk attitude, number of minors, number of elderly population, average years of education for adults, sex of household head, age of household head and squared term, and self-rated health of household head) and area characteristics variables (rural and province fixed effects). To provide the reader with a better understanding of the meaning of the variables, we have explained the critical variables in S1 Table. A trivariate score of the household financial inclusion index shows that the proportion of poor and vulnerable households decreases gradually as the level of financial inclusion increases, with 12% of poor and 37% of vulnerable families in the low financial inclusion group, 5% of poor and 22% of vulnerable households in the medium financial inclusion group, and only 2% of poor and 6% of vulnerable families in the high financial inclusion group.

**Table 3:** Descriptive statistics of variables.

Variables	Observations	Average value	Standard deviation	Minimum value	Maximum value
Financial Inclusion Index	34.449	39.155	16.02	0	100
Household Net Worth (million- RMB)	34.449	67.150	106.25	0	811.0
Household income (million- RMB)	34.449	6.25	7.02	0	60.02
Financial assets as a percentage of total assets	34.449	0.145	0.020	0	1
Financial Literacy	34.449	55.14	33.15	0	100
Relationship Network	34.449	18.145	10.20	0	100
Private lending participation	34.449	0.202	0.40	0	1
Social pension insurance participation	34.449	0.145	0.45	0	1
Per capita social pension insurance balance (million)	34.449	0.301	0.02	0	7.5
Social health insurance participation	34.449	0.02	0.46	0	1
Social health insurance balance per capita (million)	34.449	0.20	0.145	0	1.5
Preferred risk	34.449	0.21	0.14	0	1
Number of minors	34.449	0.502	0.02	0	10
Number of elderly population	34.449	0.052	3.52	0	5
Average years of schooling	34.449	9.41	0.43	0	22
Male	34.449	0.23	13.02	0	1
Age	34.449	52.052	0.12	16	80
Self-rated health	34.449	0.16	0.25	0	4

### 2.5. Method

#### 2.5.1. Demonstration method

Consider the simplest linear probability model:

$$y_{i}=x_{i}^{\prime }\beta +\varepsilon_{i}\;(i=1,\cdots ,n)$$

If ***β*** satisfies the consensus estimate, then Cov(*x*_*i*_, ε_*i*_) = 0 is required. However, since $\varepsilon_{i}=y_{i}-x_{i}^{\prime }\beta$, so $\varepsilon_{i}=1-x_{i}^{\prime }\beta$, and therefore ε_*i*_ must be related to $x_{i}^{\prime }$, the model estimates are inconsistent. Also, the variance of the disturbance term ε_*i*_ depends on $x_{i}^{\prime }$ due to $\mathrm{Var}(\varepsilon_{i})=\mathrm{Var}(x_{i}^{\prime }\beta )$, so the model has heteroscedasticity. In order to make the predicted value of *y* between 0 and 1, given the independent variable, consider the following two-point distribution.


$$\{\begin{array}{l} P(y=1|x)=F(x,\beta )\\ P(y=0|x)=1-F(x,\beta ) \end{array}$$

Since the value of the above function is either 0 or 1, it obeys a two-point distribution, and because:

E(y|x)=1⋅P(y=1|x)+0⋅P(y=0|x)=P(y=1|x)

If *F*(***x***, ***β***) is the standard normal cumulative distribution function, then:

$$P(y=1|x)=F(x,\beta )=\Phi (x^{\prime }\beta )=\int_{-\infty }^{x^{\prime }\beta }\phi (t)dt$$

This model is called the "Probit" model, and if *F*(***x***, ***β***) is the cumulative distribution function of the "logistic distribution", then:

$$P(y=1|x)=F(x,\beta )=\Lambda (x^{\prime }\beta )\equiv \frac{\exp(x^{\prime }\beta )}{1+\exp(x^{\prime }\beta )}$$

This model is called the "Logit" model.

This paper uses the Probit model. This section uses the Probit model to examine the impact of financial Inclusion on household poverty and vulnerability. The explanatory variables are the household poverty and vulnerability dummy, which are assigned a value of one of the households is poor and zero otherwise. In addition, one of the households is vulnerable and zero otherwise. The explanatory variables are household poverty dummy and vulnerability dummy, the explanatory variables are household financial inclusion index, and the control variables include household characteristics and regional characteristics variables. The model is set up as follows (1), *y*_*i*_ = 1 indicating that household i is a poor or vulnerable household, findex_i_ the household financial inclusion index, and a set of control variables.


$$\mathrm{Pt}(y_{i}=1|)findex_{i},X_{i})=\beta_{0}+\beta_{1}findex_{i}+\beta_{2}X_{i}+\varepsilon_{i}$$

#### 2.5.2. Instrumental variable method (2SLS)

Consider the simplest univariate linear regression model:

$$y_{i}=\alpha +\beta x_{i}+\varepsilon_{i}\;(i=1,\ldots ,n)$$

If there is endogeneity in the model, it means that the explanatory variable is related to the disturbance term:

$$\mathrm{Cov}(x_{i},\varepsilon_{i})\neq 0$$

Then, the explanatory variables are endogenous variables, and the endogeneity of the model may lead to inconsistent estimation results. No matter how large the sample size is, the model estimated value will not be infinitely close to the true value parameter.

The idea of instrumental variables is actually quite simple. While an endogenous variable is a "bad" variable (related to the perturbation term), there may still be a "good" part (the part that is not related to the perturbation term). If an endogenous variable can be decomposed into the sum of its endogenous and exogenous components, it is possible to obtain a consistent estimate using its exogenous component.

To achieve this separation, it is usually necessary to rely on another variable, called "Instrumental Variable" (Instrumental Variable. Abbreviation IV), because it plays an instrumental role. Obviously. Not any variable can be used as an instrumental variable. The instrumental variables themselves must be "clean", that is, "exogenous" (unrelated to perturbation terms).


$$\mathrm{Cov}(z_{i},\varepsilon_{i})=0$$

Second, the instrumental variables must also satisfy the correlation:

$$\mathrm{Cov}(z_{i},x_{i})\neq 0$$

Assuming that a suitable instrumental variable is found, it can be regressed on the dependent variable to separate out the exogenous part:

$$x_{i}=\gamma +\delta z_{i}+u_{i}$$

This regression is the first stage regression. The fitted value is ${\hat{x}}_{i}=\hat{\gamma }+\hat{\delta }z_{i}$; the residual is

$${\hat{u}}_{i}=x_{i}-{\hat{x}}_{i}.$$

This decomposes the endogenous variable into two parts:

$$x_{i}={\hat{x}}_{i}+{\hat{u}}_{i}$$

The fitted value is a linear function of the instrumental variable, so it is exogenous; and the residual part is the endogenous part. Finally, OLS is used to obtain a consistent estimate of the parameters:

$$y_{i}=\alpha +\beta {\hat{x}}_{i}+(\varepsilon_{i}+\beta {\hat{u}}_{i})$$

This regression is the second stage regression. In this way, the fitted value of the independent variable is not correlated with the disturbance term.

The explanatory variable in this paper is consumption poverty, and controlling for variables such as household income and assets in the model avoids reverse causality due to income or asset differences. However, some uncontrollable factors may still lead to endogeneity problems, such as omitting other non-financial poverty alleviation support available to households or informal financial services usage habits may lead to underestimating the impact of financial Inclusion. Given this, we use an instrumental variables approach to address the endogeneity issue. Referring to the common practice of designing instrumental variables in the literature, we mainly use the financial inclusion index of other households in the same community as the instrumental variable, denoted as "Bartik IV" [41, 42]. Financial Inclusion of other households in the community can affect the financial inclusion status of the home through co-mortgage, co-bonded loans, or private lending.

In contrast, the financial inclusion status of other households does not directly affect the poverty or vulnerability of the family, thus satisfying the basic requirements of the instrumental variable. The between-group differences of this instrumental variable across communities ensure that the estimation is technically feasible and realistic. In addition, we construct as an instrumental variable "the time horizon of financial inclusion promotion at the provincial level × the dummy variable that the mean value of the financial inclusion index of other households in the community is higher than its national mean value(Assign a value of 1 if the average value of other household financial inclusion indices in the community is higher than the national average, otherwise 0)". Provincial promotion of financial Inclusion can increase the financial Inclusion of households in the province. Local actions are relatively exogenous to micro families, so the instrumental variable satisfies the requirements of relevance and homogeneity. The instrumental variable implies that households are more likely to improve exogenously when their province promotes financial inclusion development and when the financial inclusion status of other homes in the community is higher than the national average households’ financial inclusion status.

#### 2.5.3. Fragility decomposition

Household vulnerability, on the one hand, maybe due to its risk factors (idiosyncratic shocks), and on the other hand, it may be due to external risk factors (synergistic shocks). Communities are the grassroots governance units in China, and many policies are promoted and implemented. In addition, essential public services are provided from communities to households, so we define the scope of extrinsic risks in communities. Currently, different communities in China differ significantly in terms of economic development, public services, and natural conditions. Therefore, distinguishing various sources of risk, helps to implement targeted measures in reducing household vulnerability.

Regarding its economic significance, households can rely on themselves to cope with shock events when they face idiosyncratic shocks, or they can be dealt with through co-insurance mechanisms within the community. However, when households are exposed to synergistic community shocks, where all homes in the same neighborhood may be at risk, community co-insurance mechanisms will be challenging to help families deal with them effectively, and households will have to rely more on themselves. Therefore, financial Inclusion may be an effective complement. To this end, we adopt the vulnerability decomposition method of [26], which decomposes household vulnerability into vulnerability due to idiosyncratic shocks and exposure due to synergistic shocks according to the source of risk shocks, and investigate whether financial Inclusion can help households better cope with vulnerability due to synergistic shocks. The model is set up as follows (2), using a sample of vulnerable families, *y*_*i*_ = 1 indicating that household vulnerability is due to synergistic shocks, *y*_*i*_ = 0 indicating that vulnerability is due to idiosyncratic shocks only, findex_*i*_ being the household financial inclusion index, and *X*_*i*_ being the control variable.


$$\mathrm{Pr}(y_{i}=1|{\mathrm{findex}}_{i},X_{i})=\beta_{0}+\beta_{1}f{\mathrm{index}}_{i}+\beta_{2}X_{i}+\varepsilon_{i}$$

## 3. Empirical analysis

### 3.1. Basic estimation results of financial inclusion on poverty and vulnerability

This paper uses all data from the China Household Finance Survey (CHFS) 2019, including poor and non-poor households, rural and urban households, mainly using the World Bank’s poverty line and the vulnerability indicator based on the standard definition of poverty proposed by [24]. This section uses the Probit model. This section uses the Probit model to examine the impact of financial Inclusion on household poverty and vulnerability. The explanatory variables are the household poverty dummy and the vulnerability dummy, which are assigned a value of one of the households is poor and zero otherwise. One of the households is vulnerable and zero otherwise. The explanatory variables are household poverty dummy and vulnerability dummy, the explanatory variables are household financial inclusion index, and the control variables include household characteristics and regional characteristics variables. The model is set up as follows (1), *y*_*i*_ = 1 indicating that household i is a poor or vulnerable household, findex_i_ the household financial inclusion index, and a set of control variables.


$$\mathrm{Pt}(y_{i}=1|)findex_{i},X_{i})=\beta_{0}+\beta_{1}findex_{i}+\beta_{2}X_{i}+\varepsilon_{i}$$ (1)

Using the $1.9 poverty line, Table 4 reports the regression results of financial Inclusion on household poverty and vulnerability. First, we use the time-space double fixed panel regression model for modeling, and columns (1)-(2) show the time and space dual fixed panel regression results, which add all control variables. It can be seen that when all control variables are added, the marginal effect of the financial inclusion index on household poverty and vulnerability is significantly negative at the 1% level, indicating that financial Inclusion helps reduce the probability of household poverty and vulnerability. Secondly, we did not add these variables to the regression equation because there is a certain correlation between the financial inclusion index and variables such as household income, financial assets, and pension insurance. The regression results are shown in columns (3)-(4). It can be seen from this that when the above variables are not added, the marginal effect of the financial inclusion index on household poverty and vulnerability is significantly negative at the 1% level, indicating that financial Inclusion helps reduce the probability of household poverty and vulnerability. Finally, variables such as household income, financial assets, and pension insurance are not only related to the financial inclusion index but also the poverty and vulnerability of households. If they are not controlled in the regression, there may be a problem of missing variables. Columns (5)-(6) report the complete estimation results. At this time, the marginal effect of the financial inclusion index has declined, but it is still significantly negative at the 1% level. The financial inclusion index rises by one standard deviation, and households The probability of poverty and vulnerability will dramatically drop by 1.42% and 0.63%. In addition, using the linear probability model (LPM) for estimation can also reach consistent conclusions; improving the level of financial Inclusion can significantly improve household poverty and vulnerability. Finally, the stepwise regression method is used in this paper; the control variables are gradually added to the model (1) to observe the model results, and consistent conclusions can also be obtained.

**Table 4:** Primary estimation results of financial inclusion on household poverty and vulnerability.

	Poverty	vulnerability	Poverty	vulnerability	Poverty	vulnerability
	(1)	(2)	(3)	(4)	(5)	(6)
Financial Inclusion Index	-0.0023**	-0.0020***	-0.0038***	-0.0029***	-0.0016***	-0. 0015***
	(0.0017)	(0.0016)	(0.0021)	(0.0018)	(0.0001)	(0.0002)
Log of Assets	-0.0237***	-0. 0248***			-0.0152***	-0. 0255***
	(0.0006)	(0.0033)			(0.0007)	(0.0053)
Social health insurance participation	-0.0325**	-0. 0661**			-0.0125***	-0. 0725***
	(0.0035)	(0.0027)			(0.0025)	(0.0025)
Social insurance balance per capita	-0.0038***	-0. 0465***			-0.0025**	-0. 0125***
	(0.0006)	(0.0007)			(0.0006)	(0.0007)
Other control variables	YES	YES	YES	YES	YES	YES
Provincial fixed effects	YES	YES	YES	YES	YES	YES
Observed values	34125	33725	34125	33725	34125	33725

Note

***, **, and * represent significance levels of 1%, 5%, and 10%, respectively; marginal effects are reported in the table, and standard errors of clustering at the province level are in parentheses. The estimation results of other household characteristics and regional characteristics variables are generally consistent with the literature findings, such as financial literacy and relationship networks, which help reduce household poverty and vulnerability. However, an increase in the number of dependents such as minors and the elderly, which do not help reduce household poverty and exposure, is not reported due to space constraints.

### 3.2. Results of instrumental variable estimation

The explanatory variable in this paper is consumption poverty, and controlling for variables such as household income and assets in the model avoids reverse causality due to income or asset differences. However, some uncontrollable factors may still lead to endogeneity problems, such as omitting other non-financial poverty alleviation support available to households or informal financial services usage habits may lead to underestimating the impact of financial Inclusion. Given this, we use an instrumental variables approach to address the endogeneity issue. Referring to the common practice of designing instrumental variables in the literature, we mainly use the financial inclusion index of other households in the same community as the instrumental variable, denoted as "Bartik IV" [41, 42]. Financial Inclusion of other households in the community can affect the financial inclusion status of the home through co-mortgage, co-bonded loans, or private lending.

In contrast, the financial inclusion status of other households does not directly affect the poverty or vulnerability of the family, thus satisfying the basic requirements of the instrumental variable. The between-group differences of this instrumental variable across communities ensure that the estimation is technically feasible and realistic. In addition, we construct as an instrumental variable "the time horizon of financial inclusion promotion at the provincial level × the dummy variable that the mean value of the financial inclusion index of other households in the community is higher than its national mean value(Assign a value of 1 if the average value of other household financial inclusion indices in the community is higher than the national average, otherwise 0)". Provincial promotion of financial Inclusion can increase the financial Inclusion of households in the province. Local actions are relatively exogenous to micro families, so the instrumental variable satisfies the requirements of relevance and homogeneity. The instrumental variable implies that households are more likely to improve exogenously when their province promotes financial inclusion development and when the financial inclusion status of other homes in the community is higher than the national average households’ financial inclusion status.

Table 5 reports the IV estimation results.4 Columns (1)-(2) are estimated using the Probit model, and the Bartik IV showing that the financial inclusion index is an endogenous variable and the instrumental variable t-value and the one-stage regression F-value reject the original hypothesis that the weak instrument, KPrkLM statistic, denies under-identification, confirming that the instrumental variable is appropriate. The marginal effect of the financial inclusion index increases after considering the endogeneity issue, suggesting that not considering the endogeneity issue would underestimate the impact of financial Inclusion. Further, the estimation of columns (3)-(4) using the LPM model and the Bartik IV shows that financial Inclusion significantly reduces household poverty and vulnerability. The regression coefficients are larger than when the endogeneity issue is not considered. A one standard deviation increase in the financial inclusion index will reduce the probability of poverty and vulnerability of households by 8.75% and 7.43%. Columns (5)-(6) continue to add community variables such as community disposable income per capita, the share of industrial and commercial households, the percentage of minors and elderly, infrastructure, grassroots governance, and public security to enhance the homogeneity of instrumental variables, and obtain broadly consistent findings. The estimation of columns (7)-(8) using provincial policy IV shows that financial Inclusion significantly reduces household poverty and the regression coefficients are similar to the other columns. In contrast, financial inclusion regression on vulnerability does not have significant endogeneity issues. Overall, the instrumental variable estimation shows that financial Inclusion still significantly reduces household poverty and vulnerability, while ignoring the endogeneity issue underestimates the impact of the economic implications of financial Inclusion.

**Table 5:** Estimation results of instrumental variables.

	IV-Probit		2SLS		2SLS		2SLS(Provincial Policy)	
	Poverty	vulnerability	Poverty	vulnerability	Poverty	vulnerability	Poverty	vulnerability
	(1)	(2)	(3)	(4)	(5)	(6)	(7)	(8)
Financial Inclusion	-0.018***	-0.0062***	-0.0048***	-0.0050***	-0.0056***	-0.0025*	-0.0052*	-0.0022**
	(0.0041)	(0.0058)	(0.0056)	(0.0025)	(0.0026)	(0.0022)	(0.0023)	(0.0026)
Control Variables	YES	YES	YES	YES	YES	YES	YES	YES
Community Variables					YES	YES	YES	YES
Province	YES	YES	YES	YES	YES	YES	YES	YES
Observations	33726	33726	337823	33723	336203	3353	33735	33723
DWH- *χ*^2^	77.352***	33.23***	36.63***	32.26***	26.26***	17.63***	14.23***	0.223***
*IV*-*t*	12.23***	12.323***	12.23***	12.26***	10.83***	10.23***	3.30***	3.63***
Phase I F-value	277.23***	277.323***	277.022***	277.32***	245.336***	23.30***	15.263***	15.26***
KPrkLM	407.52***	418.323***	15.230***	14.63***	14.35***	13.032***	8.263***	8.63***

### 3.3. Is financial inclusion " more flowers on the brocade" or " fuel in snowy weather"?

The purpose of financial Inclusion is to extend the outreach of financial services and provide sustainable financial services to disadvantaged groups that are financially excluded. The Plan for Promoting the Development of Inclusive Finance (2016–2020) formulated by the State Council states that farmers, low-income urban people, poor people, and special groups such as people with disabilities and the elderly are the current key service targets of inclusive finance in China. In other words, financial Inclusion’s motive is to let the disadvantaged groups enjoy the financial services they deserve, and Financial Inclusion should play the role of " fuel in snowy weather " in poverty alleviation. However, suppose financial Inclusion is only the icing on the cake and has a more favorable impact on non-disadvantaged groups. In that case, we need to be cautious about its role in the fight against poverty in China.

To this end, we study the role of financial Inclusion in poverty alleviation in China by constructing interaction terms between the financial inclusion index and related variables in rural areas, agricultural household registration, urban household income, elderly head of household, and household members with serious chronic diseases. Using the LPM model and Bartik IV for estimation, the explanatory variables are dummy variables for household poverty, and column (1) of Table 6 shows that the interaction term between the financial inclusion index and rural areas is significantly negative at the 1% level, i.e., financial Inclusion has a more significant effect on reducing poverty in rural households compared to urban households. Similarly, columns (2)-(5) show that financial Inclusion is more helpful in improving poverty among farm households, urban low-income, elderly, and households with members suffering from severe chronic diseases. Overall, financial Inclusion plays a role in reducing poverty in China by providing the financial services needed by vulnerable groups, such as rural and urban low-income households, which will help reduce their vulnerability to poverty.

**Table 6:** Whether financial inclusion is a " more flowers on the brocade" or " fuel in snowy weather" (2SLS estimates).

Variables	Explained variable: poverty				
	(1)	(2)	(3)	(4)	(5)
Financial Inclusion	-0.025***	-0.0052	-0.0152***	-0.0023***	-0.0032***
	(0.025)	(0.0022)	(0.029)	(0.002)	(0.0023)
Financial Inclusion*Rural	-0.0052***				
	(0.0015)				
Financial Inclusion*Agricultural Household Registration		-0.0023***			
		(0.0008)			
Financial Inclusion*Income			0.0023***		
			(0.0002)		
Financial Inclusion* Elderly				-0.0237***	
				(0.00023)	
Financial Inclusion*Chronic Diseases					-0.0031***
					(0.0008)
Control variables	YES	YES	YES	YES	YES
Province fixed effects	YES	YES	YES	YES	YES
Observed values	33715	33525	23052	33752	33715

Note

***, **, and * represent significance levels of 1%, 5%, and 10%, respectively; regression coefficients are reported in the table, and standard errors of clustering at the province level are in parentheses. The table has controlled for the five variables used for the interaction and other characteristic variables, and the interaction terms have been treated endogenously.

### 3.4. Robustness test

First, we tested using other poverty lines (per capita income below 2800 yuan to measure rural poverty, the low-income line to measure urban poverty, and half of the median household income in each province to measure relative poverty) and other vulnerability lines (50% and 29%) and obtained consistent findings. Second, the [43] method was used to generate household financial inclusion dummy variables by assigning and summing the financial services indicators (dummy variables take the value of 1 if the calculated score is more significant than 0.5 and 0 otherwise) and estimated using the PSM method, and the findings remain robust. Finally, considering that Local Average Treatment Effect (LATE) may lead to a several-fold increase in the IV estimated coefficient of Financial Inclusion, we refer to [44] and conduct a heterogeneity analysis of LATE through grouped regressions to reveal in which groups the impact of financial Inclusion is mainly reflected. The results show that financial Inclusion’s effects are more significant in the subsamples of rural, farming, low-income, low financial literacy, poor health, and elderly households, with relatively small changes in the IV estimated coefficients. In comparison, financial Inclusion’s impact is more minor in other subsamples such as urban. Still, the IV estimated coefficients increase substantially, leading to an increase in the IV estimates under the overall sample.

## 4. Analysis of the impact mechanism

### 4.1. Mechanisms of the impact of financial Inclusion on household poverty: Promoting entrepreneurship

The existing literature theorizes that financial Inclusion can positively impact poverty by promoting productive activities such as entrepreneurship, but mainly does not provide empirical evidence [45, 46]. The entrepreneurial channel of financial Inclusion involves the following two questions; entrepreneurial activities help reduce household poverty, and Financial Inclusion can increase entrepreneurship. Along with this logic, we test whether financial Inclusion can reduce household poverty by promoting entrepreneurial activities. Compared with the literature, we make the following two extensions: first, we divide the sample into two groups of poor and non-poor households to discuss the differential impact of financial Inclusion on entrepreneurship of different families; if financial Inclusion helps poor households to escape from poverty in the long run, then the effect on entrepreneurship of poor households should be more significant; second, we set dummy variables based on the persistence of household entrepreneurship to study the impact of entrepreneurship persistence on poverty and How financial Inclusion affects household entrepreneurial persistence. (Bruhn & Love, 2014) takes a financial accessibility perspective and finds that financial accessibility can impact household poverty status through the labor market channel.

Using Bartik IV, entrepreneurship is measured by whether the household is involved in business and industry, and the estimates are reported in Table 7. Column (1) shows that business operations help reduce the likelihood of poverty among households. Columns (2)-(4) show that financial Inclusion significantly promotes households’ participation in business operations and has a more significant positive effect on low-income families. Further, columns (5)-(6) also show that only continuing business significantly reduces household poverty, and the impact of both new business creation and exit from industry is not significant. In contrast, financial Inclusion substantially reduces the likelihood of household exit from the business. A one standard deviation increase in the financial inclusion index significantly reduces the probability of household exit from the company by 5.26 percent. Due to the short duration of business operation, the poverty alleviation effect has not yet been fully realized for start-up households. The exit of families from business operations due to capital, tax burden, or poor business performance also does not help to improve household poverty. Overall, this section shows that financial Inclusion can positively affect household poverty by promoting entrepreneurial activity, as reflected both in its more significant impact on promoting entrepreneurship among poor households and in its ability to significantly reduce the likelihood of families exiting their businesses.

**Table 7:** Tests on the mechanism of the impact of financial inclusion on household poverty (2SLS estimates).

Variables	Poverty(1)	Industrial and commercial operations			Poverty(5)	Exit Operations(6)
		National(2)	Poverty(3)	Non-poor(3)		
Business and industry operations	-0.0215***(0.0523)					
Financial Inclusion Index		0.0052***(0.0141)	0.0015**(0.0015)	0.0015***(0.0572)		-0.0521*(0.0419)
Going Concerned					-0.035(0.006)	
New Business					-0.0126(0.0524)	
Exit operations					0.0077(0.0149)	
Control variables	YES	YES	YES	YES	YES	YES
Province fixed effects	YES	YES	YES	YES	YES	YES
Observed values	33554	33515	21533	31126	33551	545363

Note

***, **, and * represent significance levels of 1%, 5%, and 10%, respectively; regression coefficients are reported in the table, and robust standard errors of clustering at the province level are in parentheses. To avoid reverse causality problems in the model, household assets and income in the control variables are subtracted from business assets and net business profits, respectively.

### 4.2. Mechanisms of the impact of financial inclusion on household vulnerability: enhancing risk management capacity

Household vulnerability is determined by the size of risk shocks on the one hand and is also constrained by the household’s risk management capacity on the other. In general, the larger the risk shock, the greater the likelihood of household vulnerability, but if homes have poor risk management capacity themselves, they are more likely to fall into poverty when faced with a risk event. Financial Inclusion, by providing households with needed financial services such as commercial insurance coverage or emergency loan support, can serve as an effective tool for families to dispose of risks and help them improve their risk management capacity [47]. Given this, this section tests whether financial Inclusion can reduce vulnerability by improving the risk management capacity of households in China. We use "pension plan approach" and "loan channel preference" to reflect households’ risk management capacity, and families have robust risk management capacity if they develop their pension plans mainly through their savings, investment, and insurance (savings pension plan); if If the household only uses children’s support, spouse’s relatives’ support, and retirement pay, then the risk management ability is considered weak. Also, under the assumption of borrowing money, risk management ability is more vital if households prefer to obtain loans through banks. In contrast, risk management ability is weaker if they use informal loans such as relatives, friends, and business partners.

Using Bartik IV, Table 8 reports the estimation results. Columns (1)-(2) show that financial Inclusion, while not having a significant effect on households’ formulation of pension plans, has a significant positive impact on savings pension plans, i.e., financial Inclusion significantly increases households’ formulation of pension plans through savings, investment, and insurance. Column (3) analyzes the effect of different pension plans on household vulnerability and finds that savings pension plans significantly reduce household vulnerability while other pension plans have no significant impact. Further, columns (4)-(5) show that financial Inclusion has a significant positive effect on households’ formal loan preferences, reducing the probability of homes falling into vulnerability. Overall, financial Inclusion can improve household vulnerability through channels that enhance households’ risk management capabilities, not only in the sense that financial Inclusion helps families to develop retirement plans through savings, investments, and insurance, but also in the sense that financial Inclusion can significantly increase households’ preference for formal loan channels.

**Table 8:** Tests on the mechanism of the impact of financial inclusion on vulnerability (2SLS estimates).

Variables	Have a retirement plan(1)	Savings and Retirement Plan(2)	vulnerability(3)	Formal Loan Preferences(4)	vulnerability(5)
Financial Inclusion Index	-0.0041(0.0014)	0.0015**(0.0015)		0.0153***(0.0058)	
Savings pension plan			-0.0115**(0.0015)		
Other pension plans			-0.00152(0.0059)		
Formal loan preference					-0.0048*(0.0002)
Control variables	YES	YES	YES	YES	YES
Provincial fixed effects	YES	YES	YES	YES	YES
Observed value	33741	12113	33752	32941	32905

Note

***, **, and * represent significance levels of 1%, 5%, and 10%, respectively; regression coefficients are reported in the table, and standard errors of clustering at the province level are in parentheses. Questions H3036, H3044, and H3045 in the CHFS questionnaire ask about household retirement plans and loan channel preferences, respectively.

## 5. Further analysis

### 5.1. Financial inclusion and precise poverty alleviation

Precision poverty alleviation is an important initiative in China’s efforts to solve the problems of mistargeting and elite capture in previous poverty alleviation efforts. What role can financial Inclusion play in China’s precise poverty alleviation, and whether it can promote precise poverty alleviation and improve the effectiveness of poverty alleviation are questions we are interested in and try to answer further? To this end, we analyze the differential impact of financial Inclusion on different poor households by poor grouping families based on "whether they belong to the government’s poverty alleviation households" and "whether they receive government transfer income," and then explain the role that financial Inclusion can play in China’s precise poverty alleviation. The analysis of the differential impact of financial Inclusion on different poor households and financial Inclusion in poverty alleviation in China. In addition to consumption poverty, we also use [48] counterfactual analysis, income poverty, and multidimensional poverty to measure household poverty, considering that poverty alleviation in China is based on income, housing, education, and health are all taken into account.

Instrumental variables were estimated using IV, and the results of the estimates are reported in Table 9. Panel A uses the $1.9 consumption poverty line and finds that financial Inclusion has no significant effect on poor households belonging to government pro-poor households and those receiving pro-poor transfer income, but significantly reduces poverty for non-government pro-poor families and those not receiving transfer income. Panel B uses counterfactual analysis to measure poverty and finds that financial Inclusion has a more significant effect on non-government pro-poor households and poor households that do not receive government transfer income. Overall, the analysis shows that financial Inclusion can play an important role in poverty reduction for poor families that are missed in poverty alleviation practices, reflecting the great potential of financial Inclusion in poverty alleviation issues. Increasing financial Inclusion can help enhance the effectiveness of poverty alleviation. If poor households that do not receive government support for poverty alleviation receive the financial services they need, they can, to a certain extent, compensate for the welfare loss caused by their failure to receive government support for poverty alleviation. Therefore, in addition to continuously improving the existing poverty alleviation mechanism, the practice of precise poverty alleviation also needs to promote the development of financial Inclusion.

**Table 9:** Financial inclusion and precision poverty reduction (2SLS estimates).

Panel A: $1.9 Poverty Line				
Variables	Poverty Alleviation Household Group(1)	Non-poor household group(2)	Access to transfer income group(3)	Not receiving transfer income group(4)
Financial Inclusion Index	-0.0015(0.0052)	-0.0155***(0.0521)	-0.00075(0.0006)	-0.0041***(0.0052)
Control variables	YES	YES	YES	YES
Provincial fixed effects	YES	YES	YES	YES
Observations	18602	19641	31852	33315
Panel B: Counterfactual Analysis				
Variables	Poverty Alleviation Household Group(5)	Non-poor household group(6)	Access to transfer income group(7)	Not receiving transfer income group(8)
Financial Inclusion Index	-0.00415*(0.0052)	-0.0015**(0.0020)	-0.0041(0.0041)	-0.0026***(0.0052)
Control variables	YES	YES	YES	YES
Provincial fixed effects	YES	YES	YES	YES
Observations	185521	19241	18215	193415

Note: (1) ***, **, and * represent 1%, 5%, and 10% significance levels, respectively; regression coefficients are reported in the table, and standard errors of clustering at the province level are in parentheses. (2) Column (1), y = 1 if sample i is a poor household and is pro-poor; y = 0 if it is not a poor household; column (2), y = 1 if the sample is a poor household but not pro-poor; y = 0 if it is not a poor household; similarly other models can be set. (3) CHFS only asks about 20,000 households for pro-poor household information, and the counterfactual analysis is also launched based on these 20,000 households, so the observations in columns (3)-(4) and Panel B are small. (4) Similar results can be obtained under income and multidimensional poverty.

### 5.2. Financial inclusion and vulnerability decomposition

Household vulnerability, on the one hand, maybe due to its risk factors (idiosyncratic shocks), and on the other hand, it may be due to external risk factors (synergistic shocks). Communities are the grassroots governance units in China, and many policies are promoted and implemented. In addition, essential public services are provided from communities to households, so we define the scope of extrinsic risks in communities. Currently, different communities in China differ significantly in terms of economic development, public services, and natural conditions. Therefore, distinguishing various sources of risk, helps to implement targeted measures in reducing household vulnerability.

Regarding its economic significance, households can rely on themselves to cope with shock events when they face idiosyncratic shocks, or they can be dealt with through co-insurance mechanisms within the community. However, when households are exposed to synergistic community shocks, where all homes in the same neighborhood may be at risk, community co-insurance mechanisms will be challenging to help families deal with them effectively, and households will have to rely more on themselves. Therefore, financial Inclusion may be an effective complement. To this end, we adopt the vulnerability decomposition method of (Günther & Harttgen, 2009), which decomposes household vulnerability into vulnerability due to idiosyncratic shocks and exposure due to synergistic shocks according to the source of risk shocks, and investigate whether financial Inclusion can help households better cope with vulnerability due to synergistic shocks. The model is set up as follows (2), using a sample of vulnerable families, *y*_*i*_ = 1 indicating that household vulnerability is due to synergistic shocks, *y*_*i*_ = 0 indicating that vulnerability is due to idiosyncratic shocks only, findex_*i*_ being the household financial inclusion index, and *X*_*i*_ being the control variable.


$$\mathrm{Pr}(y_{i}=1|{\mathrm{findex}}_{i},X_{i})=\beta_{0}+\beta_{1}f{\mathrm{index}}_{i}+\beta_{2}X_{i}+\varepsilon_{i}$$ (2)

Table 10 reports the vulnerability decomposition and regression results. Columns (1)-(3) show that household vulnerability in China today is mainly due to household-specific shocks, with a minor contribution from synergistic community shocks. Household-specific shocks lead to exposure in 27.26% of households, while community-coherent shocks lead to vulnerability in 1.56% of households. However, synergistic shocks have a relatively more significant impact on rural households than urban households. Using Bartik IV for estimation, columns (4)-(6) show that financial Inclusion can play a more positive role in coping with vulnerability due to synergistic shocks than vulnerability due to idiosyncratic shocks and that financial Inclusion has a more significant effect on rural households in coping with synergistic shocks. A one standard deviation increase in the financial inclusion index significantly reduces the probability of vulnerability due to synergistic shocks by 0.82% compared to the likelihood of exposure due to idiosyncratic shocks. Overall, when households experience vulnerability due to synergistic shocks, financial Inclusion can serve as an effective complement to co-insurance mechanisms to help families better manage risk events.

**Table 10:** Impact of financial inclusion on vulnerability to community shocks.

Panel A: Decomposition Results			
	National(1)	Rural(2)	City(3)
Community shocks (synergistic)	0.0156	0.0225	0.0015
Household shocks (idiosyncratic)	0.2726	0.3251	0.1702
Community shocks/household shocks	0.0441	0.0745	0.03415
Panel B: Regression results (2SLS estimation)			
	National(4)	Rural(5)	City(6)
Financial Inclusion Index	-0.0042*(0.0035)	-0.0046***(0.0005)	-0.0052(0.0026)
Control variables	YES	YES	YES
Province fixed effects	YES	YES	YES
Observations	6615	2852	2815

Note

***, **, and * represent significance levels of 1%, 5%, and 10%, respectively; regression coefficients are reported in the table, and standard errors of clustering at the province level are in parentheses.

## 6. Conclusions and policy implications

Based on the 2015 data of the China Household Finance Survey, this paper investigates the impact of financial Inclusion on household poverty and vulnerability by constructing a household financial inclusion index. It is found that financial Inclusion can significantly reduce the probability of poverty and vulnerability of households and that financial Inclusion can play a snowy role in poverty alleviation, i.e., it has a more positive impact on farmers, urban low-income people, and special groups such as the chronically ill and the elderly. Using the mean value of the financial inclusion index of other households in the same community as the instrumental variable, the linear probability model estimation shows that a one standard deviation increase in the financial inclusion index will reduce the household’s probability of poverty and vulnerability. The linear probability model estimates show that a one standard deviation increase in the financial inclusion index decreases the likelihood of poverty and vulnerability by 8.75 percent and 7.43 percent, respectively.

Further, it is found that financial Inclusion can reduce household poverty and vulnerability through promoting household entrepreneurship and improving risk management capacity, respectively. Among them, financial Inclusion encourages household participation in business operations and reduces the probability of household exit from the business. In contrast, continuous process significantly reduces household poverty, while the impact of withdrawal from the company and new business creation is not significant. Finally, the study finds that financial Inclusion contributes to the effectiveness of poverty reduction and can serve as an effective complement to co-insurance mechanisms to help households better deal with vulnerabilities caused by synergistic shocks.

The findings of this paper have important policy implications. First, it is necessary to construct a system of financial inclusion indicators at all levels. In addition to national and regional financial inclusion indices, consideration should be given to creating a micro household financial inclusion index, as this directly reflects micro households’ financial resource allocation status. Second, besides focusing on the current poverty situation, the government also needs to increase its attention to vulnerable families to avoid the emergence of new poor households and return to poverty; at the same time, efforts should be made to promote economic development, infrastructure improvement and modern governance in grassroots communities, especially to promote rural grassroots development, to prevent synergistic shocks from leading to the absence of co-insurance mechanisms and widespread exposure of households to vulnerability risks. Third, we will continue to improve the environment for innovation and entrepreneurship, provide financial support to small, medium, and micro enterprises using a financial inclusion approach, and combine tax and fee reductions to improve business sustainability and reduce business exit rates to ensure that entrepreneurial activities play a role in poverty eradication in the long term. Fourth, actively promote innovation in financial products and services, encourage county financial institutions to set aside funds for local use, guide commercial, financial institutions to provide support to the three rural areas, poor households, and micro and small enterprises, and accelerate the construction of a credit system and build a national credit sharing mechanism to reduce financial risks that may arise in the development of financial Inclusion.

## Supporting information

S1 Data (XLSX)

S1 Table (DOCX)
