# Supplementary material for: Does financial inclusion help alleviate household poverty and vulnerability in China?
Source: PLoS One. 2022 Oct 14;17(10):e0275577. doi: 10.1371/journal.pone.0275577 (PMC9565458; doi:10.1371/journal.pone.0275577)
Supplement: S1 Table — (DOCX) [file pone.0275577.s002.docx]

**Supplementary Table 1**

| Variables | Meaning |
| --- | --- |
| Financial literacy | The 2019 CHFS questionnaire was used to classify household financial literacy into two dimensions: financial literacy and financial literacy practices. 11 questions were selected to measure households' level of financial literacy, including "whether interest rates are calculated accurately." Factor analysis was conducted to reduce the dimensionality of the above 11 initial variables to obtain three factors. First, financial literacy was measured by the knowledge factor indicator, which was derived from three questions: "whether interest rates are calculated accurately," "whether inflation is correctly understood," and "whether investment risks are accurately identified." Three questions. The investment factor measures financial literacy and the savings factor, where the investment factor is mainly derived from the questions "whether you have a stock account," "whether you hold funds," "whether you own bank financial products. Six questions measure the investment factor: "Do you have a stock account," "Do you hold funds," "Do you own bank financial products," "Do you own internet financial products," "Do you own other financial products" and "Do you use credit cards." The savings factor is mainly derived from the two questions "Do you have a current passbook or savings card" and "Do you have RMB time deposits." The savings factor is mainly derived from the question "Do you have a current bankbook or savings card" and "Do you have an RMB time deposit" which is used to measure respondents' basic financial knowledge. |
| Relationship Networks | Data from the 2019 China Household Financial Survey (CHFS). The relationship network should cover the household's social spending, social status, and several family and friends. Therefore, I selected nine variables, including party membership, position, type of unit, gift expenditure, communication costs, transportation costs, entertainment costs, dining out costs, and several blood relatives (where party membership, position, and type of unit are measures of social status; gift expenditure, transportation costs, communication costs, entertainment costs, and dining out costs are measures of investment in social network building and maintenance; and several blood relatives is a measure of the number of relatives, etc.). (the number of relatives is a measure of solid relationships such as relatives), factor analysis was used to construct a composite indicator of relationship networks. Firstly, the nine variables were indexed for gift, communication, transport, entertainment, and eating out costs. The KMO indicator was used to check whether the nine variables were suitable for factor analysis. The first three factors were retained based on eigenvalues more significant than one. The relative weights of each of the main factors after factor rotation were then used to calculate the composite index of the relationship network. |
| Private Lending | A value of 1 is assigned to the presence of loans from non-bank formal financial institutions; otherwise, a value of 0 is set. |
| Attitudes to risk | Risk attitudes were measured based on the respondents' willingness to invest in the CHFS questionnaire. Consumers who chose high-risk, high-return projects indicated a strong desire to take risks and were assigned a value of 1. Conversely, consumers who chose projects that were not willing to take any risks indicated a conservative and weak willingness to take risks and were assigned a value of 0. |
| Household head self-assessment of health | Those who chose very healthy in the questionnaire were assigned a value of 4; those who generally chose healthily were assigned a value of 3; those who decided unhealthy were assigned a value of 2, and those who chose very harmful were given a value of 1. |
